# An observational study of the discrediting of COX-2 NSAIDs in Australia: Vioxx or class effect?

**DOI:** 10.1186/1471-2458-11-892

**Published:** 2011-11-24

**Authors:** Lynne Parkinson, Xenia Doljagore, Richard Gibson, Evan Doran, Lisa Notley, Jenny Stewart Williams, Paul Kowal, Julie E Byles

**Affiliations:** 1 Research Centre for Gender, Health and Ageing, The University of Newcastle, Newcastle, Australia

## Abstract

**Background:**

When a medicine such as rofecoxib (Vioxx) is withdrawn, or a whole class of medicines discredited such as the selective COX-2 inhibitors (COX-2s), follow-up of impacts at consumer level can be difficult and costly. The Australian Longitudinal Study on Women's Health provides a rare opportunity to examine individual consumer medicine use following a major discrediting event, the withdrawal of rofecoxib and issuing of safety warnings on the COX-2 class of medicines. The overall objective of this paper was to examine the impact of this discrediting event on dispensing of the COX-2 class of medicines, by describing medicine switching behaviours of older Australian women using rofecoxib in September 2004; the uptake of other COX-2s; and the characteristics of women who continued using a COX-2.

**Methods:**

Participants were concessional beneficiary status women from the Older cohort (born 1921-26) of the Australian Longitudinal Study on Women's Health who consented to linkage to Pharmaceutical Benefits Scheme data, with at least one rofecoxib prescription dispensed in the 12 months before rofecoxib withdrawal. A prescription was defined as one dispensing occasion. Women were grouped by rofecoxib pattern of use: continuous (nine or more prescriptions dispensed in the 12 months prior to rofecoxib withdrawal) or non-continuous (eight or less prescriptions dispensed in the 12 months prior to rofecoxib withdrawal) users. Incidence rate per 100,000 person days and incidence risk ratio described uptake of alternate medicines, following rofecoxib withdrawal. Kaplan-Meier curves described differences in uptake patterns by medicine and pattern of rofecoxib use. Patterns of use of COX-2s in the next 100 days after first COX-2 uptake were described.

**Results:**

Medicine switches and pattern of medicines uptake differed significantly depending upon whether a woman was a continuous or non-continuous rofecoxib user prior to rofecoxib discrediting. Continuous rofecoxib users overwhelmingly switched to another COX-2 and remained continuing COX-2 users for at least 100 days post-switch.

**Conclusions:**

The typical switching behaviour of this group of women suggests that the issues leading to the discrediting of rofecoxib were not seen as a COX-2 class effect by prescribers to this high use group of consumers.

## Background

NSAIDs (Non-Steroidal Anti-inflammatory Drugs)have been consistently associated with adverse gastrointestinal (GI) and renal effects [1]. Selective COX-2 inhibitors (COX-2s) that promised to minimize adverse GI effects [1] were first marketed in Australia in 1998, and listed on the national medicines subsidy Pharmaceutical Benefits Scheme (PBS) from 2000. While PBS guidelines suggested that COX-2s should be prescribed only to patients with a history of GI disorders, concomitant use of corticosteroids, anti-coagulants and advanced age, rather than as routine therapy [2], prescriptions for COX-2s increased rapidly, peaking at about 250,000 Australian users in 2004 [3,4], suggesting these guidelines were not being followed.

The expected advantage of fewer GI side effects for COX-2s compared to non-selective NSAIDs (ns-NSAIDs) was supported by longer-term safety studies, but early studies also showed an increase in cardiovascular (CVD) and renal events [5,6]. Subsequent studies found a fourfold increase in risk of myocardial infarction (MI) for rofecoxib (Vioxx) users compared to naproxen users (ans-NSAID) [7], an excess of CVD events in trials of COX-2 efficacy in preventing recurrent colonic polyps [8,9], and a greater risk of coronary heart disease for high-dose rofecoxib (> 25 mg/day) users in observational studies [10]. Safety concerns intensified and rofecoxib was withdrawn by the manufacturer world-wide in September 2004 [11,12]. While similar concerns were expressed in relation to other COX-2s [13], these medicines were not withdrawn. In Australia, the Therapeutic Goods Administration (TGA) required manufacturers to provide explicit product information warnings about CVD risk and advised that all medicines in the COX-2s class should be regarded as having increased CVD risk [14]. Regardless, two COX-2s, celecoxib and meloxicam, were both among the top 25 highest volume medicines dispensed on the PBS in 2006 [15]. In 2007, the TGA cancelled registration of lumiracoxib, a newly available COX-2, due to concerns about serious liver side effects, further discrediting this drug class [16]. After the withdrawal of rofecoxib, paracetamol (acetaminophen) was widely promoted as first line therapy, especially for older people with arthritis [17], given that ns-NSAIDs have also been associated with increased CVD risk [18-20].

International examination of the context of the discrediting of the COX-2s and subsequent effects on prescribing practices have shown different medicine switching patterns according to country differences in prescriber characteristics, professional recommendations and therapeutic guidelines [5,21-30].

Longitudinal evaluation of a US pharmacy claims database found increased prescribing of other NSAIDs, including those with relative COX-2 selectivity, after the withdrawal of rofecoxib and another COX-2, valdecoxib [24]. A large US nationally representative cross-sectional survey of ambulatory care visits showed relatively stable NSAIDs prescribing from 1999 to 2005; initially, COX-2s substituted ns-NSAIDs, but after withdrawal of rofecoxib, prescribing of ns-NSAIDs and non-narcotic analgesics rose sharply [28], suggesting the perception of a class effect.

In the UK, data from the General Practice Research Database showed that approximately 80% of those using COX-2s stopped within 6 months of the major discrediting event (the withdrawal of rofecoxib) [27]. In Scotland, data from a national prescription database showed that the withdrawal of rofecoxib led to a short-lived initial increase in prescription of celecoxib, and a parallel increase in the prescription of other NSAIDs, suggesting these were prescribed as alternatives to rofecoxib [21]. In Ireland, interrogation of a national prescribing database found prescribers were less likely to switch patients previously receiving rofecoxib therapy to another COX-2 irrespective of age or gender, either in the short or longer term [23]. The authors suggest that prescribers viewed rofecoxib related CVD safety concerns as a class effect [30]. However, another Irish study found evidence of chronic use of COX-2s despite discrediting [22], with female patients, those over 65 years and those at CVD risk more likely to start celecoxib rather than a ns-NSAID, suggesting that prescribers may not have perceived the discrediting as a class effect [22,30]. Other European studies had varying findings. A small Italian study found that rofecoxib withdrawal resulted in a marked decrease of COX-2 use in general practice, indicative of a belief in a class effect [26]. In contrast, a German study, using data for patients within the statutory health insurance system [29], rofecoxib withdrawal led to initial increased prescribing of other COX-2s (celecoxib, valdecoxib), similar to the situation in Scotland [21]. Subsequent safety warnings on COX-2s and the withdrawal of valdecoxib in April 2005 led to pronounced reductions in COX-2 prescribing, and increased ns-NSAID prescribing. While German physicians responded promptly to safety warnings [29], reduced prescribing of COX-2s led to a simultaneous increase in NSAID prescribing, and especially those NSAIDs with a relative preference for COX-2, which may be associated with COX-2 like safety risks [29]. In the Netherlands, a study of individual prescription patterns of COX-2 users showed that while discrediting led to a significant decrease in use of COX-2s, large numbers of patients appeared not to switch to alternative pain treatments [25].

In Australia, aggregated PBS dispensing data have been used to examine the influence of COX-2s on overall NSAIDs and paracetamol prescribing afterCOX-2 discrediting [2]. Overall NSAIDs use declined, with evidence of slight increases in the use of paracetamol in April 2005. Reduced total COX-2 prescribing following rofecoxib withdrawal suggested prescribers saw discrediting as a class effect. While these aggregated data show the rise and fall of COX-2 prescribing in Australia, individual switching behavior can only be assumed, given the lack of examination of individual level longitudinal data. A study of COX-2 use in an Australian veteran population [5], found that despite the increased vulnerability of veterans receiving heart failure or diabetes medicines to adverse NSAIDs effects, uptake rates of COX-2s were similar to the rest of the veteran population, suggesting that while GI safety messages were interpreted broadly by prescribers, other adverse effects were not considered [5]. No Australian studies have evaluated the effects of COX-2 discrediting on individual level patterns of medicine use for a large general population sample [4].

When a medicine is withdrawn, as in the case of rofecoxib, follow-up at the individual consumer level is uncommon, as it can be difficult and costly. The Australian Longitudinal Study on Women's Health (ALSWH) provides a rare opportunity to examine consumer medicine use behaviours following a discrediting event in a large cohort of older women. The overall objective of this paper was to examine the impact of the major COX-2 discrediting event (withdrawal of rofecoxib) on dispensing of the COX-2 class of medicines. The specific aims were to describe:

1. medicine switching after rofecoxib withdrawal by older Australian women using rofecoxib at30 September 2004;

2. characteristics of women who switched to an alternate COX-2 in the first 100 days after 30 September 2004; and

3. patterns of use of alternate COX-2s taken up in the first 100 days after rofecoxib withdrawal, in the next 100 days after this medicine switch.

## Methods

Data for this project are from Australian Longitudinal Study on Women's Health (ALSWH) Surveys 3 (S3) and 4 (S4) for the 1921-26 birth year cohort(undertaken 2002 and 2005), individually linked to PBS medicines data. The ALSWH is a national study which began in 1996 with a random sample of more than 40,000 women in three age cohorts [31]. Since 1998, follow-up surveys have occurred on a three yearly staggered cycle by age cohort. The Older cohort of women (*n *= 12,432) were 70-75 years at S1 in 1996 and 79-84 years at S4 (distributed March 2005, after discrediting). Retention rates over the 10 years of the study have been high (over 80% at each survey). Further information about ALSWH can be found at http://www.alswh.org.au[32]. The University of Newcastle Human Research Ethics Committee approved all aspects of the study (H-076-0795).

### ALSWH self-report survey data

The following ALSWH self-report survey variables were considered: ***Area of residence: ***urban vs. non-urban [33]; ***Ease of managing on income***. impossible/difficult all of the time/difficult some of the time, vs. not too bad/easy; ***Education***. no school qualifications, vs. school/higher qualifications; ***Marital status: ***married/living as married, vs. separated/divorced, vs. widowed/single; ***WHO Body Mass Index (BMI) ***[34]: Underweight (BMI < 18.5), vs. normal (BMI = 18.5 - 24.99), vs. overweight and obese (BMI ≥ 25.0); **Smoker**: yes, vs. no; ***Alcohol use: ***non-drinker, vs. rarely/low risk drinker, vs. risky/high risk; **General health**: excellent/very good/good, vs. fair/poor; ***Family Doctor visits in 12 months***. 0-4, vs. 5+ visits; ***Other specialist doctor visit in 12 months***. Yes, vs. no; ***Hospital doctor visit in 12 months.***Yes, vs. no; ***Self-reported conditions: ***Yes to: Arthritis (any type), Hypertension, Depression, Anxiety/Nervous, Asthma, Diabetes, Bronchitis/Emphysema, Osteoporosis, Heart Disease, Cancer diagnosed or treated in past three years?

### Pharmaceutical Benefits Scheme (PBS)medicines data

Medicare Australia collates records of prescriptions subsidised under the national PBS (including the Repatriation Pharmaceutical Benefits Scheme). These administrative data provide information about medicine item number, description, dispensing dates, costs and patient beneficiary status. Consent for linkage of PBS data was obtained from 68% of the Older cohort, with few differences between those who did and did not provide consent [35]. Details of methods used to request consent have been reported elsewhere [36]. PBS data were linked at the individual level to ALSWH survey data from 2002 to 2007.

PBS data will not include all medicines taken, particularly those that are purchased over the counter, provided in hospital, or purchased without subsidy [37]. A particular issue is that medicines costing less than the consumer "copayment" will not be captured [38]. For example, the copayment for general beneficiaries in 2002 was AUD(Australian dollars)22.40 (equivalent to USD (US dollars)40) [39], and therefore only dispensing for medicines costing more than this amount will be recorded in the PBS datasets. Where medicines of interest do fall under the copayment amount, analyses can be limited to concessional beneficiaries who pay a nominal copayment fee (e.g. AUD3.60 or USD6.43 in 2002) and for whom data capture will be complete [39].

Medicines were coded to conform to the Anatomical Therapeutic Chemical Code, to facilitate analysis [40]. The medicine groups of interest for this study were: COX-2s (including rofecoxib, celecoxib, lumiracoxib, meloxicam), ns-NSAIDs (including diclofenac, ibuprofen, naproxen, acetylsalicylic acid, piroxicam), opioids, and paracetamol(also known as acetaminophen) and combinations, defined with reference to Australian Medicines Handbook [41].

### Analyses

Women included in analyses had consented to the release of their PBS data and had a status of "concessional beneficiary" within the PBS for the entire period of follow up, as many of the medicines of interest were under copayment (as described above) for at least some of the study period. This represented 85% of the Older cohort who consented to PBS linkage.

The date of withdrawal of rofecoxib, 30 September 2004, was the start date for follow-up of medicine switching, and 31 December 2007 was the end date. Women were included only if they had at least one rofecoxib prescription dispensed in the 12 months prior to rofecoxib withdrawal (that is, 30 September 2003 to 30 September 2004), and had a minimum of 12 months follow-up (until 30 September 2005). A prescription was defined as one dispensing occasion. Each prescription for rofecoxib contained 30 tablets. Given it was expected that the switching behaviour of those with higher expressed need for these medicines (i.e., those who were dispensed them more frequently) may be different from the behaviour of those with less need, two groups of rofecoxib users were characterized, based on number of rofecoxib prescriptions dispensed [42]:

***Continuous Users***(index group for the purposes of the analysis): Nine or more rofecoxib prescriptions dispensed in the 12 months before discrediting (30 September 2003 to 30 September2004). This represents 75% or more of fulltime therapy.

**Figure 1:**
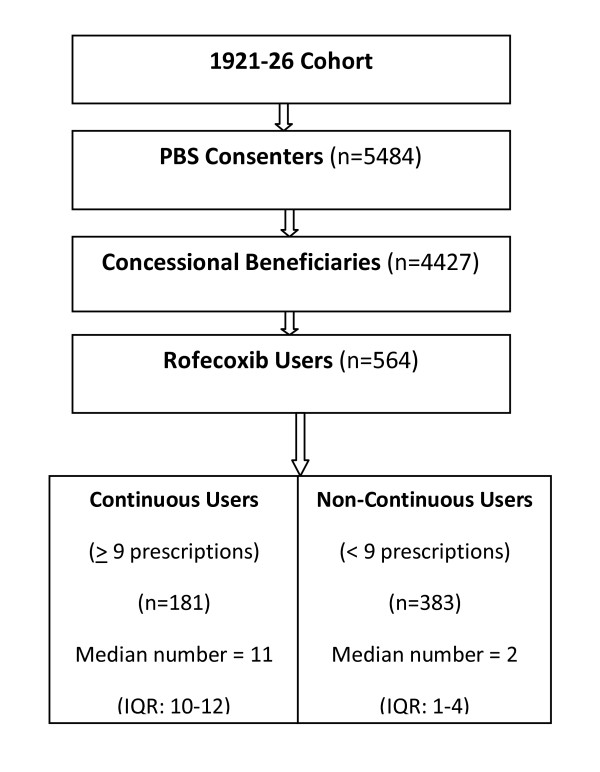
**Sample selection for ALSWH Older cohort**.

***Non-Continuous Users***(reference group): Eight or fewer rofecoxib prescriptions dispensed in the 12 months before discrediting. (See Figure 1). This non-continuous users group would be expected to have more heterogeneous prescription patterns than the continuous users group, with patterns potentially ranging from one prescription (at either end of the 12 months period), to regular prescriptions over the 12 months of interest. Median number of rofecoxib prescriptions and interquartile range was calculated for each rofecoxib user group.

The incidence rate for uptake of an alternate medicine (switching) for each rofecoxib user group was calculated as the number of incident cases divided by the total number of participant days [42,43]. Number of participant days was measured from starting date (30 September 2004) until either first prescription for the alternate medicine, the last known date of the woman in the database or the end of the study period (31 December 2007), whichever came first. Incidence risk ratios (IRR) and 95% confidence intervals (CI) were calculated for rofecoxib continuous (index) and rofecoxib non-continuous (reference) groups. Alternate medicines for rofecoxib included in these analyses were other COX-2s, ns-NSAIDs, opioids, paracetamol and paracetamol combinations, decided from formative analyses of switching behaviours (data not presented). Only women who had not been dispensed a prescription for the relevant alternate medicine within the 12 months before rofecoxib withdrawal were counted in the incidence rate. Kaplan-Meier curves were constructed for all medicines and significance determined using the log-rank test (*p *< 0.05). Only the graphs for the most common medicines switched to (meloxicam, celecoxib, and paracetamol/paracetamol combinations)are presented in this paper.

Women were further characterised as ***COX-2 switchers ***(i.e. switched to another COX-2 within 100 days after rofecoxib withdrawal) and ***COX-2 stoppers ***(did not switch to another COX-2 within 100 days after withdrawal of rofecoxib). The pattern of COX-2 use was further described for *COX-2 switchers *for the first 100 days after switching to the alternate COX-2. A *continuing *COX-2 user was defined as having been dispensed another two or more COX-2 prescriptions in the first 100 days after first prescription for an alternate COX-2 (that is, at least three prescriptions in 100 days including first prescription).

ALSWH survey demographic, health behaviour and health service use characteristics were compared for continuous rofecoxib users versus non-continuous rofecoxib users, and for COX-2 switchersversusCOX-2 stoppers, using chi square and Fishers exact tests. A conservative alpha value of 0.005 was used, due to the risk of Type1 error due to multiple comparisons. All analyses were undertaken using SAS statistical software (version 9.2) [44].

## Results

### Participants

The sample comprised 564 women including 181(33%) continuous rofecoxib users in the 12 months prior to rofecoxib withdrawal. The median number of rofecoxib prescriptions dispensed for continuous users was 11 (IQR: Q1 = 10; Q3 = 12), and 2 prescriptions (IQR: Q1 = 1; Q3 = 4) for non-continuous users. (See Figure 1).

In Table 1, continuous rofecoxib users, in the 12 months prior to rofecoxib withdrawal, were significantly more likely to report having ever been diagnosed with arthritis than were non-continuous users (94% compared to 75%, *p *< 0.001).

**Table 1:** Characteristics of non-continuous and continuous rofecoxib users (in the 12 months prior to rofecoxib withdrawal)

**Characteristic**^**c**^		**Non-Continuous rofecoxib users**^**a **^ (N = 383) %	**Continuous rofecoxib users**^**b **^ (N = 181) %	**Χ**^**2**^**, df, *p*-value**
Urban Area		51	50	0.06, 1, 0.81

Manage on Income		81	75	2.96, 1, 0.09

Education (No School)		73	71	0.12, 1, 0.73

Marital	Married/De facto	34	35	
		
	Sep/Divorced	4	6	1.81, 2, 0.40
		
	Widowed/Single	56	59	

BMI	Underweight	2	1	
		
	Normal	47	43	0.043^d^
		
	O/weight	50	56	

Smoker		4	6	0.89, 1, 0.34

Alcohol	Non-drinker	69	59	
		
	Rarely/Low drinker	30	34	0.24^d^
		
	Risky/High Risk	1	7	

Exc/V. Good/Good General Health		62	59	0.29, 1, 0.59

Consulted Family Doctor ≥5 Visits		73	77	0.94, 1, 0.33

Hospital Doctor Visit in 12 months		22	24	0.35, 1, 0.56

Specialist Doctor Visit in 12 months		61	51	4.73, 1, 0.03

Ever Reported Arthritis		75	94	**31.02, 1, < 0.001**

Conditions:	Hypertension	45	38	2.62, 1, 0.11
	
	Depression	6	9	1.14, 1, 0.28
	
	Anxiety/nervous	7	8	0.18, 1, 0.68
	
	Asthma	8	14	3.99, 1, 0.05
	
	Diabetes	11	15	1.55, 1, 0.21
	
	Bronchitis/Emphysema	6	9	1.92, 1, 0.17
	
	Osteoporosis	29	34	1.35, 1, 0.25
	
	Heart Disease	21	30	5.23, 1, 0.02
	
	Cancer	6	4	0.96, 1, 0.33

^a ^Eight or fewer rofecoxib prescriptions dispensed in 12 months before rofecoxib withdrawal (30 September 2003 to 30 September 2004)

^b ^Nine or more rofecoxib prescriptions dispensed in 12 months before rofecoxib withdrawal

^c ^See methods for details of these self-reported ALSWH variables

^d ^Fisher's exact test used due to small cell sizes, *p*-value only reported

### Medicine switching following discrediting

Continuous rofecoxib users were significantly more likely to switch to another COX-2 (IRR = 4.6; 95%CI: 3.6-5.9) or to a paracetamol based medicine (IRR = 1.8; 95%CI 1.3-2.5) than were non-continuous rofecoxib users (See Table 2).

**Table 2:** Population at risk (N), incident cases, incidence rate and IRR, by medicine and rofecoxib user status, for ALSWH Older cohort

	Continuous Users			Non-Continuous Users			IRR (95% CI)
		
Medicine	**N**^**a**^	Cases	Incidence rate (per 100,000 person days)	**N**^**a**^	Cases	Incidence rate (per 100,000 person days)	
COX-2s Overall	163	130	272.2	320	146	58.8	4.6 (3.6,5.9)

Celecoxib	163	59	44.9	320	54	16.7	2.7 (1.8,4)

Lumiracoxib	163	12	6.7	320	17	4.7	1.4 (0.6,3.2)

Meloxicam	163	98	111.3	320	100	34.8	3.2 (2.4,4.3)

ns-NSAIDs Overall	137	56	47.9	274	101	40.7	1.2 (0.8,1.6)

Diclofenac	137	14	9.6	274	24	8.0	1.2 (0.6,2.4)

Ibuprofen	137	10	6.6	274	15	4.9	1.4 (0.5,3.2)

Naproxen	137	10	6.6	274	8	2.6	2.6 (0.9,7.5)

Piroxicam	137	43	33.2	274	95	37.9	0.9 (0.6,1.3)

Acetylsalicylic acid	137	4	2.6	274	3	1.0	2.7 (0.5,18.4)

Other ns-NSAIDs	137	5	3.2	274	9	2.9	1.1 (0.3,3.7)

Opioids Overall	143	45	32.5	321	86	26.7	1.2 (0.8,1.8)

Buprenorphine	143	5	3.0	321	12	3.2	0.9 (0.3,2.9)

Morphine	143	5	3.0	321	19	5.1	0.6 (0.2,1.6)

Oxycodone	143	15	9.3	321	33	9.2	1 (0.5,1.9)

Tramadol	143	36	25.5	321	52	15.5	1.7 (1,2.6)

Other Opioids	143	2	1.2	321	4	1.1	1.1 (0.1,7.9)

Paracetamol Overall	71	56	138.0	171	96	76.6	1.8 (1.3,2.5)

Paracetamol and combinations excl. psycholeptics	71	56	138.0	171	96	76.4	1.8 (1.3,2.5)

Codeine, combinations excl. psycholeptics	71	3	3.7	171	6	3.1	1.2 (0.2,5.6)

^a ^Includes only women not dispensed these medicines in the 12 months prior to rofecoxib withdrawal

**Figure 2:**
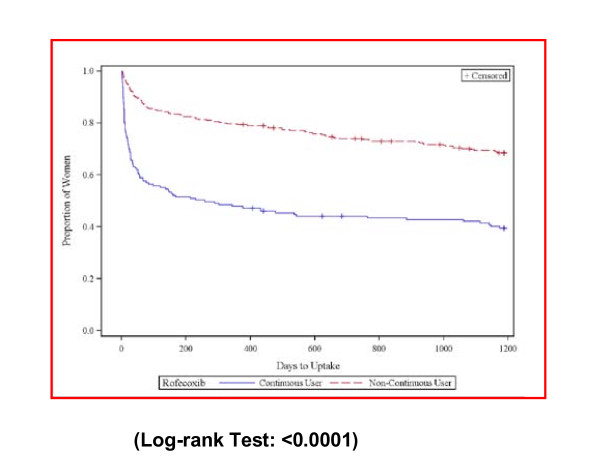
**Time to first meloxicam prescription uptake for continuous and non-continuous users**.

**Figure 3:**
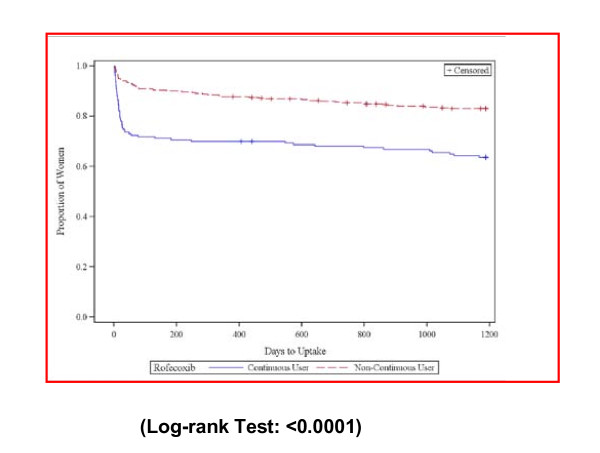
**Time to first celecoxib prescription uptake for continuous and non-continuous users**.

**Figure 4:**
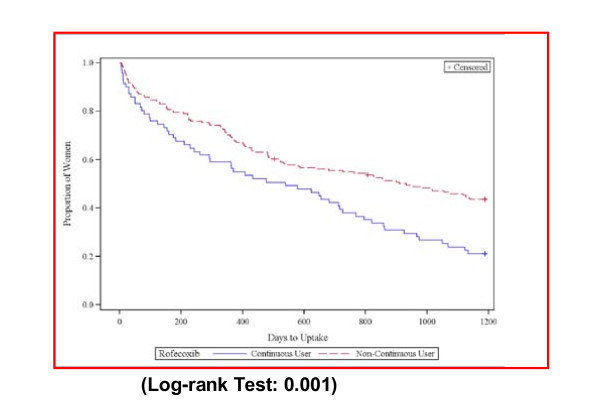
**Time to first paracetamol (combined)prescription uptake for continuous and non-continuous users**.

The Kaplan-Meier curves for uptake of meloxicam, celecoxib, and paracetamol show significantly different uptake patterns for continuous rofecoxib users compared to non-continuous rofecoxib users for all these medicines. At 100 days after discrediting, 44% of continuous users and 15% of non-continuous users had switched to meloxicam; 28% of continuous users and 9% of non-continuous users had switched to celecoxib (See Figures 2 and 3); while24% of continuous users and 15% of non-continuous users had taken up paracetamol/paracetamol combinations at 100 days after rofecoxib withdrawal (See Figure 4). At 200 days after rofecoxib discrediting, 49% of continuous users and 18% of non-continuous users had switched to meloxicam; 30% of continuous users and 10% of non-continuous users had switched to celecoxib, only small increases in uptake for both COX-2s compared to the 100 day levels. However, 33% of continuous users and 21% of non-continuous users had taken up paracetamol/paracetamol combinations at 200 days (increases of 9% and 6%), showing that uptake of paracetamol based medicines was more sustained over time than for the COX-2s.

### Characteristics of COX-2 switchers compared to COX-2 stoppers

Women who took up another COX-2(COX-2 switchers) were significantly more likely to have been a continuous rofecoxib user (47% compared to 14%, *p *< 0.0001), and to have a diagnosis of arthritis (86% compared to 77%, *p *= 0.003), than were COX-2 stoppers. (See Table 3).

**Table 3:** Demographic, health, and health care use characteristics of COX-2 switchers and COX-2 stoppers in the first 100 days after discrediting of rofecoxib

Characteristic		**COX-2 Switchers**^**a **^**(N = 189)** %	**COX-2 Stoppers**^**a **^ (N = 81) %	**χ**^**2**^**, df, *p*-value**
Continuous Rofecoxib User		47	14	**61.90, 1, < 0.0001**

Urban Area		57	45	7.54, 1, 0.01

Able to Manage on Income		80	78	0.47, 1, 0.49

No Formal Education		72	73	0.03, 1, 0.87

Marital	Married/De facto	40	36	
		
	Sep/Divorced	4	6	2.29, 1, 0.32
		
	Widowed/Single	56	58	

BMI	Underweight	1	3	
		
	Normal	44	48	0.02^b^
		
	O/weight	55	49	

Alcohol	Non-drinker	63	68	
		
	Rarely/Low drinker	32	31	0.05^b^
		
	Risky/High Risk	5	1	

Smoker		4	5	0.77, 1, 0.38

Excellent/Very Good/Good General Health		63	59	0.60, 1, 0.44

Consulted Family Doctor ≥5 Visits		70	79	5.15, 1, 0.02

Hospital Doctor Visit in 12 months		19	26	3.36, 1, 0.07

Specialist Doctor Visit in 12 months		55	62	2.56, 1, 0.11

Conditions:				

Ever Reported Arthritis		86	77	**9.03, 1, 0.003**

Hypertension		61	54	2.93, 1, 0.09

Depression		6	8	1.29, 1, 0.26

Anxiety/nervous		7	7	0.15, 1, 0.70

Asthma		9	11	0.24, 1, 0.62

Diabetes		14	11	0.93, 1, 0.33

Bronchitis/Emphysema		5	9	3.3, 1, 0.07

Heart Disease		21	27	2.16, 1, 0.14

Cancer		4	6	1.97, 1, 0.16

^a ^In the first 100 days after rofecoxib discrediting

^b ^Fisher's exact test used due to small cell sizes, *p*-value only reported

### Medicine use by COX-2 switchers in the *next *100 days after switching

**Figure 5:**
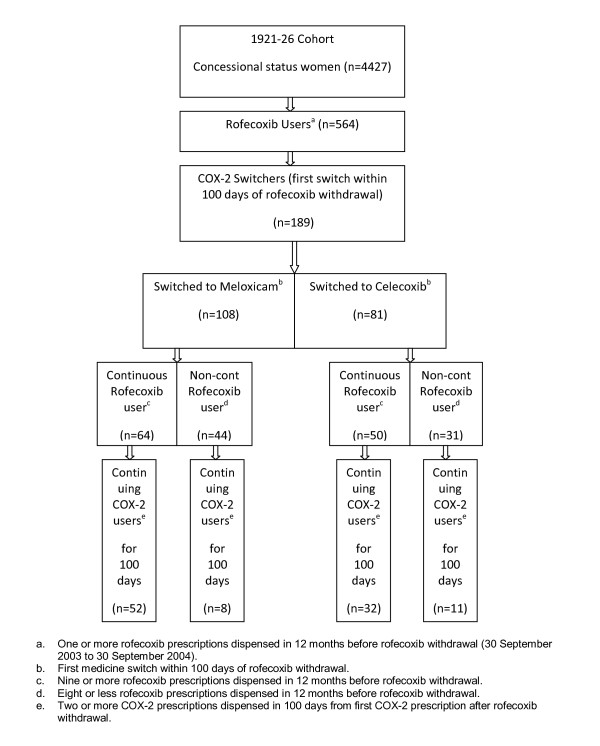
**COX-2 use in the next 100 days after first COX-2 switch**. **a **One or more rofecoxib prescriptions dispensed in 12 months before rofecoxib withdrawal (30 September 2003 to 30 September 2004). **b **First medicine switch within 100 days of rofecoxib withdrawal. **c **Nine or more rofecoxib prescriptions dispensed in 12 months before rofecoxib withdrawal. **d **Eight or less rofecoxib prescriptions dispensed in 12 months before rofecoxib withdrawal. **e **Two or more COX-2 prescriptions dispensed in 100 days from first COX-2 prescription after rofecoxib withdrawal.

In the first 100 days after rofecoxib discrediting, 189 women switched to another COX-2. Of the 108 women who switched to meloxicam, 64 had been continuous rofecoxib users and 52 became continuing COX-2 users for at least 100 days; 8 of the 44 non-continuous rofecoxib users became continuing COX-2 users. Of the 81 women who switched to celecoxib, 50 had been continuous rofecoxib users and 32 maintained continuing COX-2 use for at least 100 days; 31 had been non-continuous rofecoxib users, and 11 became continuing COX-2 users (See Figure 5).

## Discussion

This study explored individual patterns of medicine use and characteristics of COX-2 users following the withdrawal of rofecoxib (Vioxx) from the market, for women with a "concessional beneficiary" status in a national general population sample of older Australian women. The type of medicine switched to and the pattern of medicine use differed significantly depending upon whether a woman was a continuous(high use) or non-continuous (lower use) rofecoxib user in the 12 months prior to the discrediting event. Continuous users overwhelmingly switched to another COX-2 and remained a continuing COX-2 user for at least 100 days post-switch. The switching behaviour observed here suggests that the safety issues leading to the discrediting of rofecoxib may not have been seen as a COX-2 class effect by this group of Australian prescribers and consumers.

Although there has already been considerable exploration of this discrediting event [2,21-24,26-29], the strength and interest of this study is that it examines medicine use at the individual consumer level. The linkage of national subsidised pharmaceutical database medicines data to longitudinal survey data avoids the issues of consumer recall and allows examination of dispensing patterns in relation to the demographic, health and health service use characteristics of consumers. This study is novel in Australia.

There are some limitations to the study. First, the cohort includes only women. However, prevalence of arthritis is significantly higher for women than for men in the USA [45], Canada [46], the UK [47], and Australia [48,49], so this is an appropriate group in which to study the use of arthritis medicines. Second, many of the alternate medicines cost less than the PBS general copayment for at least some of the study period, meaning that only women with a "concessional beneficiary" PBS status for all years of interest could be included. This represents 85% of the Older ALSWH cohort who agreed to PBS linkage. Previous analyses have found that Older ALSWH concessional women were quite similar to non-concessional Older ALSWH women - although less likely to see a specialist or be overweight and more likely to have hypertension [35]. A third issue is that the PBS does not capture over-the-counter analgesics, so use of these medicines may be underestimated. However, in other research, where PBS data was compared with medicines data collected from a home visit medicine audit for people aged 65 years and over, PBS data was found to be close to complete, so we might expect the same for this group of concessional status older women [50]. A fourth issue is that the patterns of prescriptions for the non-continuous users group, included as a comparator for the continuous users group, are acknowledged as potentially more heterogeneous than for the continuous users group. The non-continuous users group is defined as women dispensed eight or fewer rofecoxib prescriptions in the 12 months before discrediting, on the premise that these women had a lesser expressed need for these medicines. So, this group could potentially include women who received only one prescription early in the 12 months (where the discrediting event was unlikely to be an issue), or one prescription late in the 12 months, or regular prescriptions over the 12 months of interest. The median number of scripts in this group is two prescriptions (IQR: 1-4) in 12 months, so there would be few women who received regular prescriptions, but time of dispensing could be quite varied. This means that interpretation of behaviour for this group must be circumspect, and may not necessarily be closely tied to the discrediting event. This group is useful, however, as a comparator for the switching behaviour of continuous users.

The patterns of medicine switching varied significantly depending upon whether a woman was a continuous or non-continuous rofecoxib user pre-discrediting. Continuous rofecoxib users overwhelmingly switched to another COX-2, mostly within 100 days of rofecoxib withdrawal, and the majority remained continuing COX-2 users for at least another 100 days. When an alternate COX-2 was not taken up in the first 100 days by erstwhile continuous rofecoxib users, they were less likely to be taken up over the 1200 days of the study. Interestingly, a significant proportion (24%) of non-continuous rofecoxib users also became continuing COX-2 users for at least 100 days after first post-discrediting uptake; however a direct switch to another COX-2 was much less common for this group of less frequent rofecoxib users. It appears from these findings that rofecoxib discrediting was not seen as a class effect by many prescribers to these women. This contrasts with the findings of Barozzi & Tett who suggested that reduced COX-2s prescribing following rofecoxib withdrawal confirmed prescribers saw the discrediting as a class effect [2]. While this previous analysis showed that the rise and fall of the COX-2s did markedly influence overall NSAIDs prescribing in Australia, this was only at an aggregate level, and individual switching behaviour could only be assumed. As shown in the current study, at the individual level, it may be that consumer characteristics and preference played a part in the decision on switching. Certainly, those who had previously been dispensed rofecoxib less frequently before discrediting, those who might be assumed to have a lesser need, were less likely, then to take up another COX-2, but this was not the case for those with higher expressed need, those more frequently dispensed rofecoxib. In an aggregate analysis, this detail can be lost, if there are considerably fewer continuous than non-continuous users.

There were few individual characteristics associated with early uptake of an alternate COX-2. COX-2 switchers were more likely to have a diagnosis of arthritis and to have been a continuous rofecoxib user than were COX-2 stoppers, both these circumstances potentially indicating women who experienced more persistent pain. There is some evidence that consumer satisfaction with the COX-2s is high [51], so women may have preferred to stay with this drug class. Non-continuous users may indeed have only used rofecoxib on very few occasions(as evidenced by a median number of only two prescriptions dispensed in 12 months), so rofecoxib withdrawal may have had only minimal impact on their medicine use behaviour.

There is general international clinical agreement that paracetamol should be the pain killer of first choice for arthritis, as a full therapeutic dose provides adequate analgesia for many people, with less risk of side-effects or interactions compared with NSAIDs and other analgesics [2,3,52-54]. New PBS listings for paracetamol containing preparations since April 2005 allow for use of higher doses and quantities of paracetamol specifically for chronic arthropathies and arthritis pain, with the intention of encouraging use of appropriate, regular doses of paracetamol [2]. In this study we found that continuous rofecoxib users were more likely to switch to paracetamol than were non-continuous users. Uptake of paracetamol was slower, and more sustained, and given this slow uptake, the link with the discrediting event may be somewhat tenuous. Again, the findings of Barozzi & Tett [2] that use of paracetamol was steady across 1997-2004 with a slight increase in April 2005, can be contrasted with the current individual level data, which provides the nuances available at this level of data. (See Figure 4).

## Conclusions

While the risk profile that lead to the withdrawal of rofecoxib might be indicative of aCOX-2 class effect [11,19,55], this may not have been the understanding of many prescribers (or consumers) for this group of community living older Australian women. This interpretation of COX-2 class risk has also varied across international studies of prescribing following the rofecoxib discrediting event, with some studies demonstrating a prescriber response to a class effect [2,23,26,28,30], and some not [22,24,26]. Further research is appropriate to confirm our findings, given this controversy. There is some evidence for continuing chronic use of COX-2s despite discrediting [22], and it would be of interest to explore if this is the case for this group of women, and consider the potential impact on longer term health outcomes.

## Abbreviations

ALSWH: Australian Longitudinal Study on Women's Health; CI: confidence interval; COX-2: Cyclooxygenase-2 inhibitor; CVD: cardiovascular disease; GI: gastrointestinal; IQR: Inter Quartile Range; IRR: Incidence risk ratios; NSAID: Non-Steroidal Anti-inflammatory Drug; ns-NSAID: non-selective Non-Steroidal Anti-inflammatory Drug; PBS: Pharmaceutical Benefits Scheme; S1: Survey 1; S3: Survey 3; S4: Survey 4; vs: versus

## Competing interests

The authors declare that they have no competing interests.

## Authors' contributions

LP: conceived of the study, and lead its design and coordination, and lead drafting of the manuscript; XDG: participated in the design of the study, carried out the statistical analyses and provided critical review of the manuscript; RG: participated in the design of the study, advised on and conducted formative statistical work, and provided critical review of the manuscript; ED: participated in design of the study and provided critical review of the manuscript; LN: participated in the design of the study, and provided critical review of the manuscript; JSW: participated in the design of the study, and provided critical review of the manuscript; PK: participated in the design of the study, and provided critical review of the manuscript; JEB: conceived of the study, and participated in its design and provided critical review of the manuscript. All authors read and approved the final manuscript.

## Pre-publication history

The pre-publication history for this paper can be accessed here:

http://www.biomedcentral.com/1471-2458/11/892/prepub
